# Incidence, risk factors and risk prediction of hospital-acquired suspected adverse drug reactions: a prospective cohort of Ugandan inpatients

**DOI:** 10.1136/bmjopen-2015-010568

**Published:** 2017-01-20

**Authors:** Ronald Kiguba, Charles Karamagi, Sheila M Bird

**Affiliations:** 1 Department of Pharmacology and Therapeutics, Makerere University College of Health Sciences, Kampala, Uganda; 2 Clinical Epidemiology Unit, Makerere University College of Health Sciences, Kampala, Uganda; 3 Medical Research Council Biostatistics Unit, Cambridge, UK

**Keywords:** CLINICAL PHARMACOLOGY, EPIDEMIOLOGY, GENERAL MEDICINE (see Internal Medicine), GYNAECOLOGY

## Abstract

**Objectives:**

To determine the incidence and risk factors of hospital-acquired suspected adverse drug reactions (ADRs) among Ugandan inpatients. We also constructed risk scores to predict and qualitatively assess for peculiarities between *low-risk* and *high-risk* ADR patients.

**Methods:**

Prospective cohort of consented adults admitted on medical and gynaecological wards of the 1790-bed Mulago National Referral Hospital. Hospital-acquired suspected ADRs were dichotomised as *possible* (possible/probable/definite) or *not* and *probable* (probable/definite) or *not*, using the Naranjo scale. Risk scores were generated from coefficients of ADR risk-factor logistic regression models.

**Results:**

The incidence of *possible* hospital-acquired suspected ADRs was 25% (194/762, 95% CI: 22% to 29%): 44% (85/194) experienced serious *possible* ADRs. The risk of *probable* ADRs was 11% (87/762, 95% CI 9% to 14%): 46% (40/87) had serious *probable* ADRs. Antibacterials-only (51/194), uterotonics-only (21/194), cardiovascular drugs-only (16/194), antimalarials-only (12/194) and analgesics-only (10/194) were the most frequently implicated. Treatment with six or more conventional medicines during hospitalisation (OR=2.31, 95% CI 1.29 to 4.15) and self-reported herbal medicine use during the 4 weeks preadmission (OR=1.96, 95% CI 1.22 to 3.13) were the risk factors for *probable* hospital-acquired ADRs. Risk factors for *possible* hospital-acquired ADRs were: treatment with six or more conventional medicines (OR=2.72, 95% CI 1.79 to 4.13), herbal medicine use during the 4 weeks preadmission (OR=1.68, 95% CI 1.16 to 2.43), prior 3 months hospitalisation (OR=1.57, 95% CI 1.09 to 2.26) and being on gynaecological ward (OR=2.16, 95% CI 1.36 to 3.44). More drug classes were implicated among *high-risk* ADR-patients, with cardiovascular drugs being the most frequently linked to *possible* ADRs.

**Conclusions:**

The risk of hospital-acquired suspected ADRs was higher with preadmission herbal medicine use and treatment with six or more conventional medicines during hospitalisation. Our risk scores should be validated in large-scale studies and tested in routine clinical care.

**Strengths and limitations of this study**

- A pilot was conducted to assess the feasibility of the cohort and to refine study tools.
- Medical and gynaecological inpatients were recruited and followed-up.
- The need to collect high-quality data limited the number of inpatients studied to 762.
- Clinical examination was the main method used to identify suspected adverse drug reactions due to limitations in timely availability of laboratory investigation results.

## Background

Adverse drug reactions (ADRs) are ranked the fourth and seventh leading cause of death in the USA and Sweden, respectively.^1^ ^2^ A cohort of 3322 inpatients in the UK showed a 16% incidence of hospital-acquired ADRs, but a German study of 907 inpatients with a large amount of laboratory data and parameters of vital status at baseline reported an ADR incidence of 38%.^3^ ^4^ A recent systematic review of European inpatients, including 13 studies, reported a 10.1% median percentage incidence of hospital-acquired ADRs.^5^ However, a lower incidence (6%) of ADRs was reported in a South African cohort of 665 medical inpatients.^6^

Known risk factors for ADRs include patient-related (age, gender, genetic make-up and pregnancy), drug-related (number of administered conventional medicines, drug class's toxicity profile, eg, antiretroviral therapy (ART) and drug dose), disease-related (eg, comorbidities, chronic/acute condition) and social (alcoholism, smoking, use of alternative/herbal medicines—hereafter herbal medicines) characteristics.^4^ ^7–9^ The ADR risk factors for a patient population can be used to create ADR risk-prediction models for use in routine clinical practice to identify at-risk patients for ADRs.^10^ ^11^ A good risk-prediction model should undergo four stages, namely development (to identify risk factors for designing the model) and validation (test model performance) in the first instance, and subsequently, impact (usefulness in routine clinical practice) and implementation (acceptance for use in clinical practice) assessment.^11^ However, little is known about the incidence of hospital-acquired ADRs^6^ and their risk factors among inpatients in sub-Saharan Africa, particularly in Uganda, which the present study aimed to determine. We also sought to describe the characteristics of the hospital-acquired suspected ADRs and identify the commonly implicated drug classes; and to construct ADR risk-prediction models for *possible*/*probable* ADRs to differentiate between *low-risk* and *high-risk* ADR-patients, particularly on characteristics not used to compute the risk scores.

## Methods

### Study design and setting

This prospective cohort study was conducted among adult hospitalised patients (≥18 years) at the 1790-bed Mulago National Referral Hospital with at least 140 000 inpatients annually. Details of the study setting, cohort design, data collection and data management have been described elsewhere.^12^ Briefly, three medical wards: Infectious Diseases and Gastrointestinal Illnesses (IDGI); Haematology, Neurology and Endocrinology (HNE); and Cardiovascular, Pulmonology and Nephrology (CPN); and one Gynaecological ward (GYN) constituted our study setting. Each ward has an official bed capacity of 54 but can receive up to 80 inpatients. Total daily admissions on each of the IDGI and CPN medical wards average 10–15 patients and 5–10 patients in HNE, thus 25–40 medical ward admissions per day. The GYN ward admits 20–25 patients per day.

### Data collection

During October to November 2013, a pilot study was conducted on the wards to assess the feasibility of the main cohort and to pretest the study tools. Data obtained from the pilot study, however, are not included in the final analyses. The main study started in December 2013 to April 2014 when research teams recruited and followed-up inpatients on the study wards using a systematic random sampling procedure whereby three new admissions per day on long-stay wards (HNE/CPN) and six per day on short-stay wards (IDGI/GYN) were to be recruited. Each ward-team purposed to select at random one of the first two (IDGI), three (HNE) and four (CPN/GYN) new admissions, and there after every second, third and fourth admission, respectively.

Four research teams collected the data from inpatients who had voluntarily given written informed consent. Each team comprised a medical doctor, pharmacist and degree nurse. Prior to data collection, the data collectors received week-long training on the practical pharmacovigilance aspects of the study including how to detect suspected ADRs using trigger tools. The principal author conducted daily reviews of study procedures to ensure adherence to the study protocol. A gynaecologist/obstetrician and an internist, both staff of Mulago Hospital based on the gynaecological and medical wards, respectively, were the study physicians who resolved any clinical problems encountered by the data collection teams, while the senior clinical pharmacist (principal author) resolved pharmacological issues.

Each research team conducted the baseline patient assessment to obtain relevant data on demographics, clinical conditions and medications used and thereafter conducted daily assessments until discharge, transfer, death or loss to follow-up. In each research team, clinical data related to suspected ADRs were captured from clinical notes in the patient's file, from clinical examination of the patient by each team's medical officer and by patient/caregiver/ward staff interviews. Each research team's pharmacist interviewed the patient at recruitment or used the patient's available medical documents to obtain baseline information on any medications used in the 4 weeks preceding hospitalisation. Medication data were obtained from the patient's clinical notes, treatment sheets, drug administration charts, dispensing records of ward pharmacies, pill count validation of a patient's oral medication (tablets, capsules) and by viewing of unused injectable medicines in the possession of the patient/caretaker; and by daily interviews with the patient/caregiver or ward staff to elicit further information on clinical signs and symptoms not documented in the patient's records. Research teams collected data daily from 08:00 to 18:00 from Monday to Friday and from 10:00 to 18:00 on weekends and public holidays.

### Data management

We used Epidata V.3.1 software for data entry and applied check programmes to limit out-of-range data entry errors. To verify the quality of data abstraction and entry, 10% of the 762 case report forms (CRFs) were randomly re-sampled using Stata V.12.0 (StataCorp. Stata Statistical Software: Release 12. College Station, Texas: StataCorp LP; 2011) to generate 76 cases for whom previously abstracted data were re-abstracted by RK and re-entered into the Epidata V.3.1 databank by a pretrained data entry clerk. Double-data entry verification was used to identify and record discrepancies between previously entered data against data capture at the re-entry phase. Where data discrepancies occurred, the original CRF was cross-checked and the error corrected. If the estimated discrepancy rate did not exclude a 10% data entry error-rate as the upper 95% confidence limit, the principal author re-checked all 762 original records for all important such fields, which included number of *possible* suspected ADRs.

### Identification of suspected ADRs

We defined suspected ADRs according to the WHO definition.^13^ ^14^ Operationally, a *suspected ADR* was any undesirable medical occurrence that developed after the administration of a drug and for which there was, at least, a *possible* causal relationship between the drug and the event as measured by the Naranjo ADR Probability Scale.^15^ ^16^ *Possible* ADRs included possible, probable and definite ADRs while *probable* ADRs represented probable and definite ADRs only and were dichotomised to *possible* ADR or *not* and, separately, to *probable* ADR or *not*. Preventable and non-preventable suspected ADRs, as measured by the modified Schumock and Thornton Preventability Scale,^17^ ^18^ were evaluated. Severity was assessed using the Division of AIDS Table for Grading the Severity of Adult and Paediatric Adverse Events^19^ and seriousness according to the WHO Uppsala Monitoring Centre (UMC) criteria.^20^ Clinical examination was the main method used to identify suspected ADRs. To increase the probability to detect suspected ADRs, patients were screened using an ADR trigger tool.^21^ Consensus agreement on ADR causality, preventability, severity and seriousness was reached in a committee headed by the ward-based study physician and senior clinical pharmacist (principal author). We used the team approach, other than individual assessments followed by comparison of interobserver agreement, to reflect the routine on-ward practice for solving clinical problems whereby nurses, medical doctors and clinical pharmacists brainstorm on patients' clinical problems and consult relevant literature before arriving at clinical decisions.

## Statistical analysis

### Sample size estimation

With a presumed incidence of *possible* hospital-acquired suspected ADRs of around 16%, 200 inpatients would suffice for a SE of 2.5%; with 800 inpatients needed for the 95% CI to have a width of 5%.^3^ We found a substantially higher incidence of *possible* suspected ADRs (25%; 194/762) so that, in practice, the same goal was met.

### Incidence of *possible*/*probable* hospital-acquired suspected ADRs

Incidence was computed as the total number of patients who developed at least one new possible (or *probable*) hospital-acquired suspected ADR linked to the use of a drug initiated during the current hospitalisation expressed as a proportion of the total number of patients in the study cohort. We excluded all community-acquired suspected ADRs that existed at the time of hospital admission or emerged as new suspected ADRs during the current hospitalisation as a result of exposure to medication used preadmission.

### Frequency of ADR-implicated drugs and hospital-acquired suspected ADRs

Proportions of drug classes most frequently implicated for suspected ADRs were expressed, at patient-level, as the number of patients who experienced an ADR linked to a named drug class divided by the total number of patients who experienced; (i) *possible* hospital-acquired suspected ADRs and (ii) *probable* hospital-acquired suspected ADRs. At ADR-level, the proportion of hospital-acquired suspected ADRs by drug class was expressed as a percentage of the total number of hospital-acquired suspected ADRs in the cohort, whereby a patient contributed one or more ADRs. Frequencies of *possible* hospital-acquired suspected ADRs in each drug class and for each individual drug were also reported.

### Risk factors for *possible* and *probable* hospital-acquired suspected ADRs

To identify the risk factors for *possible*/*probable* hospital-acquired suspected ADR, we tested the joint effect of all potential risk factors using binary logistic regression: first on ‘experienced at least one *probable* ADR’, and subsequently on *possible* ADR. Results obtained for linearity were compared with those using categorisation by comparing the log-likelihoods for regressors in logistic regression for natural logarithm of the odds on having experienced *possible* or *probable* ADR. However, wide 95% CIs for the *probable* ADR model signalled over-fitting and so we simplified to six key explanatory variables: linear age, gender, number of conventional medicines (≥6), Charlson's comorbidity index (≥3), preadmission herbal medicine use and HIV-positive serostatus. Two additional explanatory variables (hospitalisation in previous 3 months and gynaecological ward) were tested in the *possible* ADR regression model. Prior to regression analysis, the missing-assigned approach was used to attribute low frequency missing data for categorical variables (use of herbal medicines and previous hospitalisation) to the ‘no’ stratum. Logistic regression models for *possible/probable* hospital-acquired ADRs were stratified by HIV serostatus (HIV-positive vs HIV-negative/unknown HIV serostatus) to assess for interaction. Results are reported as ORs with their corresponding 95% CIs. Owing to the multiplicity of testing for interaction between HIV-positive serostatus and the six categorized explanatory variables, interaction needed to be significant at the 1% level (Bonferroni correction).

### Risk scores for predicting actual cases of *possible* and *probable* hospital-acquired ADRs

Each patient's risk-score for developing a *possible*/*probable* hospital-acquired ADR was computed on the natural logarithmic scale using regression coefficients from the final logistic regression models for *possible*/*probable* hospital-acquired ADRs, see online supplementary appendix S1 for details of risk-score computation.

We used the risk-score extreme subsets (*low-risk* vs *high-risk*) to examine for qualitative differences between the predicted ADR cases in the *low-risk* and *high-risk* groups on characteristics which were not part of risk-score computation, namely implicated drug class, ward (for *probable* ADR only), number of working diagnoses per patient, number of hospital-acquired suspected ADRs per patient and nature of ADR, see online supplementary Appendix S1 for details of qualitative differences between the *low-risk* and *high-risk* ADR-patients.

10.1136/bmjopen-2015-010568.supp1 supplementary appendices

## Ethical clearance

Ethical approval for the study was obtained from the School of Medicine Research and Ethics Committee, Makerere University College of Health Sciences (REC REF No. 2011-113), the Mulago Hospital Research and Ethics Committee (MREC 253) and the Uganda National Council for Science and Technology (HS 1151).

## Results

### Patient characteristics

Table 1 shows characteristics of the 762 inpatients in the study cohort. The median age of inpatients was 30 years (IQR: 24–42 years) with a median length of hospital stay of 4 days (IQR: 3–6 days). Forty-two per cent of inpatients (320/762) were on the IDGI ward and 30% (232/762) were HIV-positive.

**Table 1:** Demographic and clinical characteristics of 762 hospitalised patients, Uganda, 2014

	Hospital-acquired ADRs				
	*Possible* ADR		*Probable* ADR		
Characteristics	Yes	No	Yes	No	All patients
Number of patients	194 (25)*	568 (75)	87 (11)*	675 (89)	762 (100)
Age in years (median and IQR)	29 (25–39)	30 (24–43)	29 (24–40)	30 (24–42)	30 (24–42)
Gender					
Male	51 (22)	177 (78)	22 (10)	206 (90)	228 [30]
Female	143 (27)	391 (73)	65 (12)	469 (88)	534 [70]
Length of stay in days (median and IQR)	6 (4–8)	4 (3–6)	6 (4–8)	4 (3–6)	4 (3–6)
Patient-days of hospitalisation	1230	2511	564	3177	3741
HIV-serostatus (% of total)					
Positive	56 (24)	176 (76)	28 (12)	204 (88)	232 [30]
* On antiretroviral therapy*†	*23 (19)*	*99 (81)*	*14 (11)*	*108 (89)*	*122 [53]*
* Not on antiretroviral therapy*†	*33 (30)*	*77 (70)*	*14 (13)*	*96 (87)*	*110 [47]*
Negative	98 (29)	242 (71)	38 (11)	302 (89)	340 [45]
Unknown	40 (21)	150 (79)	21 (11)	169 (89)	190 [25]
Hospitalisation in previous 3 months					
No	125 (23)	407 (77)	54 (10)	478 (90)	532 [70]
Yes	69 (30)	161 (70)	33 (14)	197 (86)	230 [30]
Use of herbal medicines in the 4 weeks prior hospitalisation					
No	127 (23)	428 (77)	52 ( 9)	503 (91)	555 [73]
Yes	67 (32)	140 (68)	35 (17)	172 (83)	207 [27]
Ward					
Infectious diseases and gastrointestinal illnesses	58 (18)	262 (82)	30 ( 9)	290 (91)	320 [42]
Haematology, neurology and endocrinology	21 (18)	96 (82)	11 ( 9)	106 (91)	117 [15]
Cardiovascular, pulmonology and nephrology	49 (37)	85 (63)	21 (16)	113 (84)	134 [18]
Gynaecology	66 (36)	125 (64)	25 (13)	166 (87)	191 [25]

Italics represents further stratification of data in the ‘Positive’ stratum under HIV-serostatus.

*Incidence of *possible* hospital-acquired ADRs was 25% (194/762; 95% CI 22% to 29%): 85 (44%) of 194 patients experienced serious *possible* ADRs; and 11% (87/762; 95% CI 9% to 14%) *probable* ADRs: 40 (46%) of 87 patients had serious *probable* ADRs.

†Test of significance for developing an ADR among HIV-positive patients using (vs not using) antiretroviral therapy; χ^2^_(df=1)_=3.93; p=0.048 for *possible* ADR and χ^2^_(df=1)_=0.09; p=0.770 for *probable* ADR.

( )=row %; [ ]=column %.

ADRs, adverse drug reactions.

### Incidence of hospital-acquired suspected ADRs

The incidence of *possible* hospital-acquired suspected ADRs was 25% (194/762; 95% CI: 22% to 29%): 85 (44%) of 194 patients experienced serious *possible* ADRs. The risk of *probable* ADRs was 11% (87/762; 95% CI: 9% to 14%): 40 (46%) of 87 patients had serious *probable* ADRs. HIV-positive patients on ART had a significantly lower incidence of *possible* hospital-acquired ADRs (19% vs 30%; p=0.048) but no difference when *probable* ADRs were assessed (11% vs 13%; p=0.770) (see table 1).

### Frequency and characteristics of hospital-acquired suspected ADRs

A total of 344 *possible* hospital-acquired suspected ADRs were experienced by 194 inpatients or 101 *probable* hospital-acquired suspected ADRs were encountered by 87 inpatients (see tables 2 and 3). Ten per cent (33/344) of *possible* hospital-acquired suspected ADRs were identified from laboratory and other objective investigations. Gastrointestinal (46%) and neurological (23%) disorders were the commonest system organ classes affected (see table 3). Fifty-five per cent (188/344) of *possible* hospital-acquired suspected ADRs were probably or definitely preventable, 13% (45/344) severe or life-threatening and 31% (106/344) serious; none were fatal (see table 4). Frequencies of the *possible* hospital-acquired suspected ADRs, by drug class and individual pharmacological agents, are shown in table 5.

**Table 2:** Drug classes most frequently ADR-implicated among the 194 inpatients with possible hospital-acquired ADRs and the 87 inpatients with probable hospital-acquired ADRs, Uganda, 2014

		Hospital-acquired ADRs, n (%)	
Drug class	All ADRs, n (%)	*Possible* ADR	*Probable* ADR
Single drug class			
Antibacterials (Antibact) only	61 (19)	51 (26)	12 (14)
Antiretrovirals (ART) only	38 (12)	0 ( 0)	0 ( 0)
Uterotonics only	26 ( 8)	21 (11)	5 (6)
Cardiovascular (CVS) drugs only	21 (7)	16 (8)	7 (8)
Antimalarials (Antimal) only	19 (6)	12 (6)	9 (10)
Analgesics only	12 (4)	10 (5)	7 (8)
Antituberculous (AntiTB) drugs only	8 (3)	2 (1)	0 (0)
Blood only	6 (2)	5 (3)	1 (1)
Hypoglycaemics only	6 (2)	1 (1)	0 (0)
Antifungals only	3 (1)	3 (2)	1 (1)
Herbal medicines only	2 (1)	0 (0)	0 (0)
Two or more drug classes			
Antibact and ART	17 (5)	3 (2)	2 (2)
Antibact and analgesic	13 (4)	12 (6)	7 (8)
ART and AntiTBs	5 (2)	1 (1)	1 (1)
ART+Antibact+AntiTB	4 (1)	1 (1)	0 (0)
Antibact+AntiTB	3 (1)	3 (2)	1 (1)
Central Nervous System	3 (1)	3 (2)	3 (3)
Antibact+Uterotonic	3 (1)	3 (2)	2 (2)
Antibact+Antimal	2 (1)	2 (1)	2 (2)
ART and analgesic	2 (1)	2 (1)	2 (2)
Other	66 (21)	43 (22)	25 (29)
Total	320 (100)	194 (100)	87 (100)

ADR, adverse drug reaction.

**Table 3:** System organ class distribution of 344 possible hospital-acquired ADRs in 194 inpatients and 101 probable hospital-acquired ADRs in 87 inpatients, Uganda, 2014

SOC name	*Possible* hospital-acquired ADRs, n (row %)	*Probable* hospital-acquired ADRs, n (row %)
Gastrointestinal disorders	157 (46)	46 (46)
Neurological disorders	79 (23)	23 (23)
Body—general disorders	36 (10)	5 (5)
Cardiovascular disorders	25 (7)	10 (10)
Vascular, bleeding and clotting disorders	13 (4)	5 (5)
Skin and appendages disorders	8 (2)	0 ( 0)
Others	26 (8)	12 (12)
Total	344 (100)	101 (100)

ADR, adverse drug reaction; SOC, system organ class.

**Table 4:** Causality, preventability, severity and seriousness of 344 hospital-acquired suspected ADRs experienced by 194 inpatients, Uganda, 2014

Assessment	Category	Hospital-acquired ADRs (n, %), N=344
*Causality and preventability*		
Causality	Definite	9 (2)
	Probable	92 (27)
	Possible	243 (71)
Preventability	Definitely preventable	8 (2)
	Probably preventable	180 (52)
	Not preventable	156 (45)
*Severity and seriousness*		
Severity*	Mild	148 (43)
	Moderate	151 (44)
	Severe	42 (12)
	Life-threatening	3 (1)
Serious	Yes*	106 (31)
	Required intervention to prevent damage†	54 (51)
	Caused or prolonged hospitalisation†	30 (28)
	Other medically significant condition†	14 (13)
	Other	8 (8)
	No*	238 (69)

*Denominator used was the total number of hospital-acquired suspected ADRs, n=344.

†Denominator used was the number of serious hospital-acquired suspected ADRs, n=106.

**Table 5:** Drug classes and individual drugs most frequently implicated in causing the 344 possible hospital-acquired suspected ADRs among 194 inpatients, Uganda, 2014

Pharmacological drug class	Number of hospital-acquired ADRs, n (%)*	Rank by causative drug	Rank by frequency of use	Implicated drugs (number of linked ADRs)	Hospital-acquired ADRs (number of ADRs)
Antibacterials	150 (44)	1	1	Ceftriaxone (93), metronidazole (42), levofloxacin (15), ciprofloxacin (11), azithromycin (6), erythromycin (6), amoxicillin (3), co-trimoxazole (2), ampicillin (2), cloxacillin (1), gentamicin (1), clavulanic acid (1)	Vomiting (32), dizziness (19), fever (16), nausea (15), appetite loss (14), headache (11), dizziness (8), diarrhoea (8), pruritus (6), abdominal pain (6), malaise (4), diarrhoea (4), dizziness (3), epigastric pain (3), constipation (2), skin rash (2), tachycardia (2), flatulence (1), jaundice (1), decreased urine output (1), oral sores (1), blurred vision (1), dyspepsia (1), peripheral neuropathy (1), abdominal discomfort (1), convulsions (1), paraesthesia (1), hypertension (1), palpitations (1)
Uterotonics	43 (13)	2	3	Misoprostol (36), oxytocin (10)	Lower abdominal pain (13), vaginal bleeding (11), headache (5), diarrhoea (4), dizziness (3), vomiting (2), malaise (1), palpitations (1), back pain (1), raised pulse (1), nausea (1)
Cardiovascular drugs	39 (11)	3	4	Captopril (14), carvedilol (11), nifedipine (11), frusemide (10), hydralazine (5), digoxin (3), amlodipine (3), labetalol (2), cardiac aspirin (1), lisinopril (1), propranolol (1)	Headache (8), diarrhoea (3), dizziness (3), palpitations (3), vomiting (3), epigastric pain (3), dry cough (2), abdominal pain (2), oedema (2), joint pain (1), blurred vision (1), dysuria (1), nausea (1), constipation (1), low diastolic blood pressure (1), fever (1), paraesthesia (1), burning sensation (1), malaise (1), constipation (1), orthostatic hypotension (1), hypovolaemia (1)
Analgesics	36 (10)	4	2	Tramadol (19), diclofenac (8), morphine (4), codeine (1), fentanyl (1) ibuprofen (1), paracetamol (1)	Vomiting (11), dizziness (6), constipation (5), epigastric pain (4), nausea (3), headache (2), tachycardia (1), palpitations (1), diarrhoea (1), hypertension (1), abdominal pain (1), pruritus (1)
Antimalarials	26 (8)	5	5	Quinine (22), artesunate (5), artemether (3), lumefantrine (3)	Dizziness (4), vomiting (4), tinnitus (3), headache (3), nausea (2), vomiting (2), palpitations (1), taste disturbance (1), appetite loss (1), raised pulse (1), epigastric pain (1), diarrhoea (1), blurred vision (1), vaginal bleeding (1), lower abdominal pain (1), dysuria (1)
Central nervous system drugs	18 (5)	6	6	Haloperidol (10), diazepam (3), phenytoin (2), benztropine (1), lignocaine (1), metoclopramide (1), atropine (1)	Dizziness (3), drowsiness (2), poor orientation to time and place (1), uncoordinated movement (1), blurred vision (1), appetite loss (1), hypotension (1), stiff neck (1), swollen tongue (1), fever (1), tremors (1), hypertension (1), swollen lips (1), malaise (1), vomiting (1), paraesthesia (1), headache (1)
Antituberculous drugs	15 (4)	7	7	Isoniazid (14), pyrazinamide (14), rifampicin (13), ethambutol (10)	Vomiting (3), diarrhoea (2), appetite loss (2), yellow eyes (1), nausea (1), fever (2), abdominal discomfort (1), paraesthesia (1), shortness of breath (1), dizziness (1)
Blood	13 (4)	8	8	Blood (13)	Vomiting (4), difficulty in breathing (2), headache (2), chills (2), hypertension (1), bleeding (1), fever (1)
Antifungals	9 (3)	9	8	Amphotericin B (4), fluconazole (4), ketoconazole (1)	Vomiting (1), malaise (1), hypokalaemia (1), nausea (1), skin rash (1), headache (1), hypertension (1), loss of vision (1)
Contraceptives	8 (2)	10	10	Ethinylestradiol (7), levonorgestrel (7)	Headache (2), excessive salivation (1), palpitations (1), vomiting (1), nausea (1), abdominal fullness (1)

*A patient may have experienced one or more *possible* hospital-acquired suspected ADRs.

ADR, adverse drug reaction.

### Frequency of drugs implicated for hospital-acquired suspected ADRs

*Possible* ADRs linked to antibacterials-only accounted for the largest proportion (26%, 51/194) of *possible* hospital-acquired ADRs attributed to a single drug class, at the patient level, followed by suspected ADRs linked to uterotonics-only (11%, 21/194), cardiovascular drugs-only (8%, 16/194), antimalarials-only (6%, 12/194) and analgesics-only (5%, 10/194), among others (see table 2). Ceftriaxone (48/51), misoprostol (20/21), nifedipine (7/16), quinine (10/12) and tramadol (7/10), respectively, were the commonest individual pharmacological agents linked to *possible* ADRs in each of the above-mentioned drug classes. Table 2 also shows that no *possible*/*probable* hospital-acquired suspected ADRs linked to antiretrovirals-only and/or herbal medicines-only were identified.

### Risk factors for *possible*/*probable* hospital-acquired suspected ADRs

Treatment with six or more conventional medicines during hospitalisation (OR=2.31, 95% CI 1.29 to 4.15) and self-reported use of herbal medicines in the 4 weeks prior hospitalisation (OR=1.96, 95% CI: 1.22 to 3.13) were the risk factors for a *probable* hospital-acquired suspected ADR (see table 6).

**Table 6:** Risk factors for *probable* hospital-acquired suspected ADRs in 87 of 762 inpatients, Uganda, 2014

	*Probable* ADR, n (%)		Crude analysis			Adjusted analysis			
Characteristics	Yes	No	OR	95% CI for OR	p Value	β-adj	OR	95% CI for OR	p Value
Gender									
Male	22 (10)	206 (90)	1.0				1.00		
Female	65 (12)	469 (88)	1.3	0.78 to 2.16	0.317	0.291	1.34	0.79 to 2.25	0.273
Age: mean (SD)	34.6 (15.8)	34.8 (14.7)	0.97	0.98 to 1.01	0.888	−0.010	1.01	0.97 to 1.01	0.269
Number of conventional medicines									
Five or less	16 (7)	228 (93)	1.0				1.00		
Six or more	71 (14)	447 (86)	2.3	1.29 to 3.98	0.005	0.838	2.31	1.29 to 4.15	0.005
Use of herbal medicines in the 4 weeks prior hospitalisation									
No	52 (9)	503 (91)	1.0				1.00		
Yes	35 (17)	172 (83)	2.0	1.24 to 3.12	0.004	0.673	1.96	1.22 to 3.13	0.005
HIV-positive									
No or unknown	59 (11)	471 (89)	1.0				1.00		
Yes	28 (12)	204 (88)	1.1	0.68 to 1.77	0.708	0.317	0.73	0.41 to 1.31	0.288
Charlson's comorbidity index									
Two or fewer	60 (11)	507 (89)	1.0				1.00		
Three or more	27 (14)	168 (86)	1.4	0.83 to 2.21	0.218	0.505	1.66	0.88 to 3.13	0.119
Intercept						−2.807	0.06	0.03 to 0.14	

Except for gender (male coded 1 and female coded 2), indicator variables were coded 0 for baseline, 1 otherwise; regression χ^2^=20.36 on 6 df (p∼0.0024); β-adj=adjusted regression coefficient.

ADR, adverse drug reaction.

Risk factors for a *possible* hospital-acquired suspected ADR were: treatment with six or more conventional medicines (OR=2.72, 95% CI: 1.79 to 4.13), self-reported use of herbal medicines in the 4 weeks prior hospitalisation (OR=1.68, 95% CI: 1.16 to 2.43), hospitalisation in the 3 months prior hospital admission (OR=1.57, 95% CI 1.09 to 2.26) and being on the gynaecological ward (OR=2.16, 95% CI: 1.36 to 3.44, see table 7).

**Table 7:** Risk factors for *possible* hospital-acquired suspected ADRs in 194 of 762 inpatients, Uganda, 2014

	*Possible* ADR, n (%)		Crude analysis				Adjusted analysis		
Characteristics	Yes	No	OR	95% CI for OR	p Value	β-adj	OR	95% CI for OR	p Value
Gender									
Male	51 (22)	177 (78)	1.0				1.01		
Female	143 (27)	391 (73)	1.3	0.88 to 1.83	0.201	0.011	1.00	0.67 to 1.53	0.959
Age: mean (SD)	33.5 (13.6)	35.3 (15.1)	1.0	0.98 to 1.00	0.151	−0.0006	1.00	0.98 to 1.01	0.933
Number of conventional medicines									
Five or less	39 (16)	205 (84)	1.0				1.00		
Six or more	155 (30)	363 (70)	2.2	1.52 to 3.32	<0.001	1.001	2.72	1.79 to 4.13	<0.001
Use of herbal medicines in the 4 weeks prior hospitalisation									
No	127 (23)	428 (77)	1.0				1.00		
Yes	67 (32)	140 (68)	1.6	1.13 to 2.29	0.008	0.519	1.68	1.16 to 2.43	0.006
HIV-positive									
No/unknown	138 (26)	392 (74)	1.0				1.00		
Yes	56 (24)	176 (76)	0.9	0.63 to 1.29	0.580	−0.589	0.55	0.31 to 0.99	0.045
Charlson's comorbidity index									
Two or less	147 (26)	420 (74)	1.0				1.00		
Three or more	47 (24)	148 (76)	0.9	0.62 to 1.32	0.614	−0.613	0.54	0.24 to 1.24	0.146
Interaction: Charlson's index≥3 & HIV+status									
No interaction	157 (25)	472 (75)	1.0				1.00		
Interaction	37 (28)	96 (72)	1.2	0.76 to 1.76	0.492	1.235	3.44	1.22 to 9.71	0.020
Hospitalisation in past 3 months									
No	125 (24)	407 (76)	1.0				1.00		
Yes	69 (30)	161 (70)	1.4	0.99 to 1.97	0.059	0.448	1.57	1.09 to 2.26	0.016
Gynaecological ward									
No	128 (22)	443 (78)	1.0				1.00		
Yes	66 (35)	125 (65)	1.8	1.28 to 2.61	0.001	0.771	2.16	1.36 to 3.44	0.001
Intercept	–	–				−2.19	0.11	0.06 to 0.23	

Except for gender (male coded 1 and female coded 2), indicator variables were coded 0 for baseline, 1 otherwise; regression χ^2^=53.99 on 9 df (p<0.001); β-adj=adjusted regression coefficient.

### Interaction between risk factors for *possible* hospital-acquired suspected ADRs

Interaction was observed between HIV-positive serostatus and having a score of 3 or more on Charlson's comorbidity index in patients with and those without a *possible* hospital-acquired ADR. A Charlson's comorbidity index of 3 or more among the HIV-positive patients seemed to pose a risk (OR=1.9) for a *possible* hospital-acquired ADR, but was protective (OR=0.4) among the HIV-negative/unknown serostatus patients. The regression χ^2^_(df=8)_=48.24 for the model without the interaction term (Charlson's comorbidity index ≥3 & HIV-positive serostatus) was significantly lower than the χ^2^_(df=9)_=53.99 for the full model with the interaction term on 1-degree of freedom. However, the interaction was not significant at p<0.01 as strict Bonferroni correction would have required.

### Qualitative differences between predicted ADR cases in the *high-risk* versus *low-risk* groups

Suspected ADR cases in the *high-risk* (vs *low-risk*) groups implicated more drug classes (*possible* (18 vs 8) and *probable* (12 vs 7) ADRs), had more frequent occurrence of ADRs linked to cardiovascular drugs (*possible* ADRs: (8/35) versus (1/31)) and were more common on the HNE ward (*probable* ADRs: (3/16) versus (0/14), see online supplementary appendices S1–S5).

## Discussion

### Incidence of hospital-acquired suspected ADRs

One in four patients experienced at least one *possible* hospital-acquired suspected ADR during the current admission, which is higher than the ADR incidence reported in South Africa^6^ but similar to the incidence reported in the UK.^3^ The South African and UK studies estimated the incidence of hospital-acquired ADRs by including, as in our cohort of inpatients, all *possible*, *probable* and *definite* ADRs. However, the South African study^6^ used the WHO-UMC criteria^22^ for ADR causality assessment rather than the Naranjo algorithm,^15^ as in our cohort and the UK study. Poor agreement between the WHO-UMC criteria and Naranjo algorithm is reported in the causality assessment of 913 ADRs by the same evaluator at a tertiary care hospital in India,^23^ which might partly explain the observed differences in the ADR incidence estimates: however, good agreement between the two causality assessment tools is reported in an assessment of 200 ADR forms submitted to an Indian pharmacovigilance centre.^24^ The comparable ADR incidences in our cohort and the UK setting should be interpreted cautiously, however, because the UK study used the Edwards and Aronson ADR definition while we used the WHO definition;^3^ ^13^ ^14^ ^25^ and we had a smaller amount of laboratory data on ADR-markers, which might have resulted in ADR underestimation in our setting. The incidence of *probable* hospital-acquired suspected ADRs was one in nine inpatients, which is half our estimate for a *possible* hospital-acquired suspected ADR. Incidence estimates of *probable* ADRs are less likely to include adverse reactions caused by underlying disease.^26^ However, ascertaining ADR causality of implicated drugs can be a challenge in a cohort of inpatients with multiple comorbidities, extensive preadmission medication exposure and concomitant medications. Moreover, the Naranjo algorithm is designed to assess ADR causality of a single drug and does not take into account drug–drug interactions.^15^ ^24^ By comparison, the median incidence estimate of hospital-acquired ADRs in European inpatients is 1 in 10, as assessed in a recent systematic review of 13 studies. A variety of ADR causality assessment methods were used, and the majority of the studies did not differentiate between *possible* or *probable* ADRs:^5^ of the three studies which did, their reported *possible* ADR incidence estimates were either lower than (8.4%),^27^ similar to (16%)^3^ or higher than (38%)^4^ ours.

### Serious hospital-acquired suspected ADRs

About half the inpatients with hospital-acquired suspected ADRs encountered serious ADRs, which highlights the substantial contribution of these ADRs to the patients' morbidity while in hospital. Our estimate of serious *possible* hospital-acquired suspected ADRs (11%, 85/762) is higher than the 2.1% reported in a meta-analysis of 39 American studies.^28^ However, none of the serious ADRs in our cohort were fatal. At least two to three deaths would have been expected among the 762 inpatients based on the ADR death estimates in the USA (0.32%) and UK (0.42%); which may be happenchance given the small sample size of our cohort compared with the two studies conducted in the western world.^3^ ^28^

### Implicated drug classes

Previous research among medical inpatients in sub-Saharan Africa documented antibiotics^6^ and antimalarials, mostly quinine^8^ as the leading causes of hospital-acquired ADRs. A recent survey of Ugandan healthcare professionals, who provided descriptions of ADRs they had encountered in the previous month—though of community-acquired and hospital-acquired ADRs, also ranked antibiotics and antimalarials (particularly quinine) among the most commonly implicated drugs.^29^ ^30^ Our prospective cohort also studied gynaecological inpatients and highlights the high burden of hospital-acquired ADRs linked to uterotonics—mostly misoprostol. Cardiovascular drugs contribute frequently to hospital-acquired ADRs in the western world^31^ and to community-acquired ADRs in South Africa.^6^ We document the frequent occurrence of hospital-acquired ADRs linked to cardiovascular drugs in our cohort of inpatients in a sub-Saharan African setting.

No hospital-acquired suspected ADRs linked to antiretrovirals-only and/or herbal medicines-only were identified: probably because most patients who received ART had initiated the ART prior to the current hospital admission, and because we measured self-reported herbal medicine use during the 4 weeks prior to the current hospitalisation.

### Risk factors for hospital-acquired suspected ADRs

Given the complexity of confirming ADR causality of implicated drugs among our study inpatients, most of whom had multiple prior and current drug exposures, we undertook sensitivity analyses to determine the risk factors for experiencing at least one *probable* hospital-acquired suspected ADR; and separately for a *possible* hospital-acquired suspected ADR.

Patients who had used herbal medicines in the 4 weeks preadmission were at a higher risk of developing a *possible*/*probable* hospital-acquired suspected ADR. Previous research in Uganda has associated the use of herbal medicines, particularly traditional herbal medicines, with the occurrence of liver disease^9^ and possibly with defaulting on ART.^32^ Drug interactions between western conventional and herbal medicines, if concurrently used, might moderate the occurrence of ADRs.^33^ On the other hand, prehospital use of herbal medicines might delay hospitalisation and resolution of the problem(s) that patients attempt to resolve by the use of the herbal medicines.^34^

The use of several conventional medicines is a known risk factor for ADRs.^4^ ^35^ The ADRs may arise from drug interactions attributable to synergism.^36^ Patients who receive large numbers of medicines may need to take even more medications to treat ADRs of already administered medicines^36^ such as the prescription of bisacodyl to treat morphine-linked constipation.^37^ Hospitalisation in the previous 3 months prior to the present admission was an independent risk factor for *possible* ADR, but not *probable* ADR, and might relate to ADR-prone patients with previous drug exposures and/or chronic disease state(s).

The risk of developing a *possible* hospital-acquired suspected ADR was twice as high on the GYN ward when compared with the three medical wards probably due to the exclusive use of uterotonics (mainly misoprostol) on the GYN ward, which ranked second in the group of pharmacological drug classes most frequently linked to *possible* ADRs.

The observed interaction between HIV-positive serostatus and Charlson's comorbidity index might be explained by the high score of 6 in the index assigned to HIV/AIDS.^38^ Thus, Charlson's comorbidity index might require adaptation for settings with a high burden of HIV/AIDS. Researchers in a high HIV/AIDS prevalence setting in South Africa have used the tool after excluding the score for HIV/AIDS; however, it is not known whether their revision was validated.^39^

### Use of risk scores to differentiate the characteristics of *low-risk* and *high-risk* ADR cases

We identified qualitative, though weak, differences between the *low-risk* and *high-risk* ADR cases by implicated drug class, nature of ADR and ward (for *probable* ADR only), but not by number of working diagnoses or number of ADRs per patient. The small number of events, especially for *probable* ADRs, or the more likely important influence of other unmeasured clinical or organisational factors (eg, medication errors with respect to the ADRs) might partly explain the weak prediction of the qualitative differences between *low-risk* and *high-risk* groups of ADR cases on characteristics not modelled in the logistic regression.

Low uptake of risk prediction compromises the safety of clinical care. Risk scores can be useful tools to screen for patients at high risk for ADRs, who would need more careful selection and close monitoring of their medications.^40^ Obtaining a good ADR risk-prediction model requires four stages, namely development and validation, and impact and implementation assessment. Our ADR risk-prediction models were not validated due to the small number of *probable* ADR events among the inpatients. The risk-prediction models, however, delivered in differentiating between *low-risk* and *high-risk* ADR-patients on characteristics not included in risk-score computation. Nonetheless, we acknowledge that these models should be validated, in our setting, using large-scale or periodic studies.

### Study limitations

The covariate ‘number of conventional medicines’, which accounted for the total number of western conventional medicines administered during hospitalisation, has the drawback that some conventional medicines may have been received after the occurrence of a suspected ADR. Partly for this reason, we categorised number of medicines to indicate simply if the patient had received six or more medicines, that is, many. Except for the risk factor ‘number of conventional medicines’, all the other tested potential risk factors (age, gender, HIV serostatus, history of previous hospital admission, comorbidities and self-reported use of herbal medicines) were known to have preceded the occurrence of a *possible*/*probable* ADR. Arguably, Cox proportional hazards with time-dependent covariates to track the daily changes in the number of medicines administered prior to *possible*/*probable* ADR might have been a better method of analysis. However, time to first *possible*/*probable* ADR in days for most cases was rather short and accurate tracking of diverse coadministered medicines before and after ADR recognition was unlikely, even if practicable, to have yielded commensurate insights. Clinical examination was the main method used to identify suspected ADRs due to limitations in timely availability of laboratory investigation results.

## Conclusions

The risk of developing a hospital-acquired suspected ADR was high. Preadmission use of herbal medicines and treatment with six or more conventional medicines during hospitalisation were the common risk factors for *possible*/*probable* hospital-acquired suspected ADRs. The risk scores were predictive of qualitative differences between *low-risk* and *high-risk* groups of ADR cases on characteristics not modelled in the regression analyses and should be validated and assessed for their usefulness and acceptance in routine clinical practice in our setting.
