# Supplementary material for: Incidence, risk factors and risk prediction of hospital-acquired suspected adverse drug reactions: a prospective cohort of Ugandan inpatients
Source: BMJ Open. 2017 Jan 20;7(1):e010568. doi: 10.1136/bmjopen-2015-010568 (PMC5253535; doi:10.1136/bmjopen-2015-010568)
Supplement: supplementary appendices [file bmjopen-2015-010568supp_appendices.pdf]

Appendix S1: Use of risk-scores to predict actual ADR cases

Risk-scores for predicting actual cases of *possible* and *probable* hospital-acquired ADRs

Risk-score (r_i) for patient i = ln [Odds of an ADR for patient i] = constant + $\beta_1X_1i + \beta_2X_2i + ..$ $+ \beta_nX_ni$: where β_1, β_2 to β_n are regression coefficients of the ADR risk factors X_1, X_2 to X_n in the final ADR logistic regression model, and ln is the natural logarithm.

Thus, the *probable* ADR risk-score was computed as follows;

> Risk-score for *probable* ADR = -2.807 – 0.317 (if HIV-positive) + 0.505 (if Charlson's Index $\geq$ 3) + 0.673 (if patient used herbal medicines in 4-weeks prior to admission) + 0.291 (if male) + 0.582 (if female) + 0.838 (if patient was administered six or more conventional medicines) – (0.010*age in years);

And the *possible* ADR risk-score was calculated as follows;

> Risk-score for *possible* ADR = -2.19 – 0.589 (if HIV-positive) - 0.613 (if Charlson's Index $\geq$ 3) + 0.519 (if patient used herbal medicines in 4-weeks prior to admission) + 0.011 (if male) + 0.022 (if female) + 1.001 (if patient was administered six or more conventional medicines) – (0.0006*age in years) + 1.235 (HIV-positive and Charlson's index $\geq$ 3) + 0.448 (if hospitalized in past 3-months) + 0.771 (if Gynaecological ward).

But ln [Odds of an ADR for patient i] = ln [Pi/(1-Pi)]:

And so ln [Pi / (1-Pi)] = r_i where Pi is the probability of an ADR and r_i is risk-score.

$Pi/(1-Pi) = \exp(r_i) = e^{ri}$

$Pi = e^{ri}(1-Pi)$

$Pi(1 + e^{ri}) = e^{ri}$

$Pi = e^{ri} / (1 + e^{ri})$

Pi = exp (Patient's risk-score) / [1 + (Patient's risk-score)]

Subsequently, the probability of a *possible*/*probable* ADR (Pi) was calculated using the patient's risk-score (r_i):

Probability for a *possible*/*probable* ADR = exp (Patient's risk-score) / [1 + (Patient's risk-score)].

Frequency distributions of *possible*/*probable* ADR probabilities were generated for: i) patients who developed a *possible*/*probable* ADR and; ii) for the whole sample of 762 patients. The 30th percentile of *possible*/*probable* ADR probabilities distribution among the 762 patients was marked off, and patients from list i) with at most the same probability who had nonetheless experienced a *possible*/*probable* ADR were identified (*low-risk* group). A similar method was used to identify patients whose probability of *possible*/*probable* ADR was above the 90th percentile and who had indeed developed a *possible*/*probable* ADR (*high-risk* group). The *possible*/*probable* ADR cases in the lowest *three* deciles (*low-risk* group) versus the topmost decile (*high-risk* group) were identified using Stata and qualitatively assessed for any peculiarities in certain aspects (number of implicated drug classes, ward if *probable* ADR, number of working diagnoses per patient, number of ADRs per patient and nature of *possible*/*probable* ADR) - *not* so much in the characteristics used to compute the risk-score (gender, age, use of $\geq$ 6 conventional medicines, use of herbal medicines, being HIV-positive, Charlson's co-morbidity index $\geq$ 3 and ward if *possible* ADR).

Characteristics of *probable* ADR cases predicted using logistic regression risk-scores

Fourteen patients with 22 *probable* suspected ADRs were in the *low-risk* (lowest 30%) group of the *probable* ADR risk distribution. Only one patient self-reported having used herbal medicines in the 4-weeks prior to hospitalization although no details of herbal medicines used were given. Two patients had received $\geq$ 6 conventional medicines during hospitalization and three were HIV-positive. Two patients on the IDGI ward experienced *probable* hospital-acquired suspected ADRs linked to medication administration errors: the ADRs were nausea (working diagnoses: septicaemia secondary to urinary tract infection and electrolyte imbalance) and vomiting (working diagnoses: immune-suppressed syndrome with oral candidiasis, delirium, Wernicke's encephalopathy and toxoplasmosis) linked to rapid intravenous infusions of ciprofloxacin and ceftriaxone, respectively. A patient experienced blurred vision linked to Benztropine (diagnosis: organic psychosis and resolving Lyme meningitis).

Sixteen patients with 17 *probable* suspected ADRs were in the *high-risk* (top 10%) group of the *probable* ADR risk distribution, all of whom had received $\geq$ 6 conventional medicines during hospitalization, were female, and had used herbal medicines in the 4-weeks pre-admission. Most patients did not provide details of the herbal medicines they had used or the information reported was vague [11/16; no details (7/11) and vague (4/11)]. Among the five patients with details, six herbal medicines were mentioned; Sere (*Bidens pilosa*), avocado leaves (*Persea americana*), Beet root (*Beta vulgaris)*, Father Anatoli proprietary herbal concoction, garlic mixture (*Allium sativum*), and aloe vera (*Aloe barbadensis*). A patient presented with jaundice linked to bisacodyl (working diagnosis: sickler in vasocclusive crisis).

Qualitative differences in the predicted *low-risk* vs. *high-risk probable* ADR cases

Implicated drug classes

Six of the seven ADR-implicated drug classes (antibacterials, analgesics, cardiovascular drugs, antimalarials, uterotonics, and antipsychotics) among the 14 cases in the *probable* ADR *low-risk* group were also implicated among the 16 cases in the *high-risk* group. Six additional drug classes were, nonetheless, identified in the *high-risk* group of *probable* ADR risk distribution which included antidepressants, benzodiazepines, contraceptives, laxatives, anaesthetics and antiulcer agents.

Ward

The HNE ward did not have any *probable* suspected ADR cases (0/14) in the *low-risk* group of *probable* ADR risk distribution but had three patients (3/16) in the *high-risk* group, a statistically significant difference.

Working diagnosis

No noticeable differences were observed in the number of working diagnoses per patient, by ward, in the *high-risk* vs. *low-risk* groups of *probable* ADR risk distribution at a cut-off point of $\geq$ 3 diagnoses, respectively: GYN [(0/5) vs. (3/5)]; IDGI [(7/7) vs. (1/3)]; HNE [(2/3) vs. (no cases)]; and CPN [(1/1) vs. (2/3)].

Number of *probable* ADRs per patient

The occurrence of two or more *probable* ADRs per patient was more common among patients in the *low-risk* group (6/14) versus those in the *high-risk* group (3/16) of *probable* ADR risk distribution, but this difference was not statistically significant.

Characteristics of *possible* ADR cases predicted using logistic regression risk-scores

Thirty-one patients with 49 *possible* suspected ADRs were in the *low-risk* group of the *possible* ADR risk distribution. Only four of 31 patients reported having taken herbal medicines in the 4-weeks pre-admission. One patient had used avocado leaves while the other three patients gave no details. Six (6/31) patients had received $\geq$ 6 conventional medicines during hospitalization. Eight patients were HIV-positive and 10 were on the GYN ward. A patient experienced decreased urine output after receiving ceftriaxone (working diagnosis: immune suppressed syndrome and bronchopneumonia). One case of nausea due to rapid ciprofloxacin infusion, as mentioned above in the *low-risk* group of *probable* ADR, was also observed here.

Thirty-five patients with 74 *possible* suspected ADRs were in the *high-risk* group of the *possible* ADR risk distribution, all of whom had received $\geq$ 6 conventional medicines during hospitalization. Twenty-five (25/35) patients reported having used herbal medicines in the 4-weeks pre-admission. Half (12/25) the patients who reported having used herbal medicines pre-admission provided details of the herbal medicines they had used which included: *Bidens pilosa*, beet root, avocado leaves or seed concoction, Father Anatoli proprietary herbal concoction, Emumbwa, Ekigaranga, unknown liquid, *Hoslundia opposita* (Kamunye), aloe vera, and Ekikatula herbal drink.

Qualitative differences in the predicted *low-risk* vs. *high-risk possible* ADR cases

Implicated drug classes

Seven of the eight ADR-implicated drug classes [antibacterials, analgesics, cardiovascular drugs, antimalarials, uterotonics, antifungals and antipsychotics but excluding anticholinergics (one case)] among the 31 cases in the *low-risk* group of *possible* ADR risk distribution were also implicated among the 35 cases in the *high-risk* group. However, 11 additional drug classes were identified in the *high-risk* group [blood (2), iron supplements (2)

and one case of each of the following drug classes: antifibrinolytics, antidepressants, benzodiazepines, contraceptives, laxatives, calcium supplements, antituberculous drugs, bronchodilator-xanthine derivatives, and antihistamine].

Ward

Only one patient out of 31 with *possible* suspected ADRs was predicted on the HNE ward in the *low-risk* group of *possible* ADR risk distribution and four of 35 in the *high-risk* group, but with no statistically significant difference. However, ward was one of the risk factors used to compute risk-scores for *possible* hospital-acquired suspected ADRs.

Working diagnosis

No noticeable differences were observed in the number of working diagnoses per patient, by ward, in the *high-risk* vs. *low-risk* groups of *possible* ADR risk distribution at a cut-off point of $\geq$ 3 diagnoses, respectively: GYN [(5/17) vs. (1/10)]; IDGI [(4/4) vs. (7/10)]; HNE [(1/3) vs. (0/1)]; and CPN [(3/5) vs. (6/10)].

Number of *possible* ADRs per patient

Two or more *possible* ADRs per patient were more common in the *high-risk* (18/35) vs. *low-risk* (10/31) groups of *possible* ADR risk distribution, but not statistically significant.

Nature of *possible*/*probable* ADR cases

There was little to chose, by nature of ADR, between ADR cases described in the *high-risk* vs. *low-risk* groups of *possible* ADRs, and likewise of *probable* ADRs. However, we take note of the more frequent occurrence of cardiovascular ADRs in the *high-risk* groups of *possible* [top 10% (8/35) vs. lowest 30% (1/31)] and, less impressively, *probable* [top 10% (2/16) vs. lowest 30% (1/14)] ADRs.

Summary of qualitative differences between ADR cases in the *high-risk* vs. *low-risk* groups

Subtle qualitative differences were observed in the characteristics of the predicted *high-risk* vs. *low-risk* ADR cases with a similar trend for both *possible* and *probable* hospital-acquired suspected ADRs. *High-risk* groups implicated more drug classes [*possible* (18 vs. 8) and *probable* (14 vs. 7) ADRs], had more cardiovascular ADRs, and more ADR cases on the HNE ward [(4/35) vs. (1/31) for *possible* ADRs – though used in computing the risk-score; (3/16) vs. (0/14) for *probable* ADRs]; but with no noticeable difference in the number of working diagnoses per patient and the number of ADRs per patient. Furthermore, the herbal medicines used by the actual ADR cases were seldom specified.

Appendix S2: Characteristics of 14 patients with *probabl e* hospital-acquired suspected ADRs in the lowest 30% (low-risk group) of *probable* ADR risk distribution, Uganda, 2014

Probability- *probable* ADR	age	gender	ward	No of drugs administered	Herbal medicines	charlson's index	HIV-positive	Hospitalization	Working diagnosis	*Probable* ADR	Implicated drug	Causality
0.069	28	Female	GYN	Less than six drugs	No	3+	Positive	No	MISSED ABORTION, INCOMPLETE ABORTION, CORPUS LUTEAL CYST	DIARRHOEA	AMPICLOX	Probable
										LOWER ABDOMINAL PAIN	MISOPROSTOL	Probable
0.058	28	Female	GYN	Less than six drugs	No	Two or less	Negative or unknown	No	BLIGHTED OVUM	DIARRHOEA	MISOPROSTOL	Probable
0.060	25	Female	GYN	Less than six drugs	No	Two or less	Negative or unknown	No	MALARIA IN PREGNANCY	TINNITUS	QUININE	Probable
										DIZZINESS	QUININE	Probable
										PALPITATIONS	QUININE	Probable
0.062	20	Female	GYN	Less than six drugs	No	Two or less	Negative or unknown	Yes	MALARIA IN PREGNANCY	TINNITUS	QUININE	Probable
0.060	24	Female	GYN	Less than six drugs	No	Two or less	Negative or unknown	No	MALARIA IN PREGNANCY	VAGINAL BLEEDING	QUININE	Probable
										LOWER ABDOMINAL PAIN	QUININE	Probable
0.055	34	Female	GYN	Less than six drugs	No	Two or less	Negative or unknown	No	MISSED ABORTION	DIARRHOEA	MISOPROSTOL	Probable
										VAGINAL BLEEDING	MISOPROSTOL	Probable
										LOWER ABDOMINAL PAIN	MISOPROSTOL	Probable
0.047	22	Male	IDGI	Less than six drugs	No	Two or less	Negative or unknown	No	SEPTICAEMIA SECONDARY TO URINARY TRACT INFECTION, ELECTROLYTE IMBALANCE	NAUSEA (REDUCED INFUSION RATE & ADR REDUCED)	CIPROFLOXACIN	Definite
0.058	52	Male	IDGI	Six or more drugs	No	Two or less	Positive	Yes	IMMUNE SUPPRESSED SYNDROME (ISS) WITH ORAL CANDIDIASIS, DELIRIUM, WERNICKE'S ENCEPHALOPATHY, TOXOPLASMOSIS	VOMITING - RAPID INFUSION	CEFTRIAXONE	Definite
0.068	35	Male	IDGI	Six or more drugs	No	Two or less	Positive	Yes	LIVER CIRRHOSIS, ABDOMINAL LYMPHOMA, ABDOMINAL TUBERCULOSIS	CONSTIPATION	MORPHINE	Probable
0.052	40	Female	IDGI	Less than six drugs	No	Two or less	Negative or unknown	Yes	ORGANIC PSYCHOSIS, RESOLVING LYME	TREMORS	HALOPERIDOL	Probable
										BLURRED VISION	BENZTROPINE	Probable
0.044	28	Male	IDGI	Less than six drugs	No	Two or less	Negative or unknown	Yes	ORGANOPHOSPHATE POISONING, ATTEMPTED SUICIDE	DROWZINESS	ATROPINE	Probable
0.044	29	Male	CPN	Less than six drugs	No	Two or less	Negative or unknown	No	BRONCHOPNEUMONIA, RULE OUT PULMONARY TUBERCULOSIS (PTB), LOBAR PNEUMONIA	HEADACHE	CEFTRIAXONE	Probable
0.067	52	Male	CPN	Less than six drugs	Yes	Two or less	Negative or unknown	No	LOBAR PNEUMONIA, ?PTB	DIARRHOEA	ERYTHROMYCIN	Probable
										ABDOMINAL PAIN	DICLOFENAC, ERYTHROMYCIN	Probable
0.055	85	Female	CPN	Less than six drugs	No	3+	Negative or unknown	No	HYPERTENSION, ATRIAL FIBRILLATION, ISCHAEMIC HEART DISEASE, COMPLETE HEART BLOCK	COUGH	CAPTOPRIL	Probable

Appendix S3: Characteristics of 16 patients with *probable* hospital-acquired suspected ADRs in the top 10% (high-risk group) of *probable* ADR risk distribution, Uganda, 2014

Probability - *probable* ADR	age	gender	ward	No of drugs administered	Herbal medicines	charlson's index	HIV-positive	Hospitalization	Working diagnosis	*Probable* ADR	Implicated drug	Causality
0.223	25	Female	GYN	Six or more drugs	Yes	Two or less	Negative or unknown	No	INEVITABLE ABORTION, INCOMPLETE ABORTION	VOMITING	CEFTRIAXONE	Probable
0.221	26	Female	GYN	Six or more drugs	Yes	Two or less	Negative or unknown	No	INCOMPLETE ABORTION, MENORRHAGIA	VAGINAL BLEEDING	MISOPROSTOL	Definite
0.225	24	Female	GYN	Six or more drugs	Yes	Two or less	Negative or unknown	Yes	POST OPERATIVE SEPSIS, RUPTURED ECTOPIC	GENERAL BODY WEAKNESS	LEVOFLOXACIN, DIAZEPAM	Probable
										HEADACHE	LEVOFLOXACIN, CEFTRIAXONE, HALOPERIDOL	Probable
0.228	22	Female	GYN	Six or more drugs	Yes	Two or less	Negative or unknown	Yes	SEVERE ANAEMIA, SECONDARY MENORRHAGIA	HEADACHE	MICROGYNON	Probable
0.218	28	Female	GYN	Six or more drugs	Yes	Two or less	Negative or unknown	No	CHRONIC LEAKING ECTOPIC PREGNANCY, THREATENED ABORTION	PARAESTHESIA	LIGNOCAINE	Probable
0.210	85	Female	IDGI	Six or more drugs	Yes	3+	Negative or unknown	Yes	ACUTE EXACERBATION OF PEPTIC ULCER DISEASE (PUD), HPERTENSIVE HEART DISEASE, RECURRENT PUD	HEADACHE	NIFEDIPINE	Probable
0.199	40	Female	IDGI	Six or more drugs	Yes	Two or less	Negative or unknown	No	URINARY TRACT INFECTION, DIABETES MELLITUS TYPE 2 (DM), MALARIA	NAUSEA	CIPROFLOXACIN	Definite
0.250	29	Female	IDGI	Six or more drugs	Yes	3+	Positive	Yes	HIV COMPLICATED WITH CRYPTOCOCCAL MENINGITIS (CCM), TUBERCULOSIS (TB)	VOMITING	TRAMADOL	Probable
0.188	47	Female	IDGI	Six or more drugs	Yes	Two or less	Negative or unknown	No	ACUTE ON CHRONIC PANCREATITIS, RULE-OUT (R/O) DM, CHRONIC PUD, CHRONIC PANCREATITIS	VOMITING	TRAMADOL	Probable
0.253	60	Female	IDGI	Six or more drugs	Yes	3+	Negative or unknown	No	MALARIA, PUD, SEPTICAEMIA, GASTRITIS	FEVER	CEFTRIAXONE, ESOMEPRAZOLE	Probable

**Probability - *probable* ADR**	**age**	**gender**	**ward**	**No of drugs administered**	**Herbal medicines**	**charlson's index**	**HIV-positive**	**Hospitaliz ation**	**Working diagnosis**	***Probable* ADR**	**Implicated drug**	**Causality**
0.222	45	Female	IDGI	Six or more drugs	Yes	3+	Positive	Yes	IMMUNOSUPRESSED SYNDROME (ISS), CHOLELITHIASIS, STEATOSIS OF LIVER, TB IMMUNE RECONSTITUTION INFLAMMATORY SYNDROME (IRIS), ACUTE LIVER INJURY, DRUG-INDUCED HEPATITIS,SOME DEHYDRATION, VIRAL HEPATITIS, SEPSIS, CCM	HYPOVOLAEMIA	FRUSEMIDE	Probable
0.227	75	Female	IDGI	Six or more drugs	Yes	3+	Negative or unknown	No	HYPERTENSIVE URGENCY, SEPTICAEMIA, R/O MALARIA, ELECTROLYTE IMBALANCE	NAUSEA	NIFEDIPINE, ESOMEPRAZOLE	Probable
0.260	56	Female	HNE	Six or more drugs	Yes	3+	Negative or unknown	Yes	HYPERGLYCAEMIA, ACUTE GASTROENTERITIS, PUD, MALARIA	HYPERTENSION	AMITRYPTYLLINE	Probable
0.212	32	Female	HNE	Six or more drugs	Yes	Two or less	Negative or unknown	Yes	ANAEMIA, MALARIA, ? UPPER GASTROINTESTINAL BLEEDING	VOMITING	LUMARTEM, CIPROFLOXACIN	Probable
0.232	20	Female	HNE	Six or more drugs	Yes	Two or less	Negative or unknown	Yes	SICKLER IN VASOCCLUSIVE CRISIS	JAUNDICE	BISACODYL	Probable
0.228	22	Female	CPN	Six or more drugs	Yes	Two or less	Negative or unknown	No	CHRONIC OBSTRUCTIVE PULMONARY DISEASE, SEPSIS, PTB, BRONCHITIS, PNEUMONITIS, HEPATITIS, BRONCHIAL ASTHMA, COR PULMONALE	EPIGASTRIC PAIN	IBUPROFEN	Probable

Appendix S4: Characteristics of 31 patients with *possible* hospital-acquired suspected ADRs in the lowest 30% (low-risk group) of *possible* ADR risk distribution, Uganda, 2014

Probability - *possible* ADR	age	gender	ward	No of drugs administered	Herbal medicines	charlson's index	HIV-positive	Hospitalization	Working diagnosis	*Possible* ADR	Implicated drug
0.196	25	Female	GYN	Less than six drugs	No	0	No	No	URINARY TRACT INFECTION (UTI) IN PREGNANCY, THREATENED ABORTION	HEADACHE	TRAMADOL
0.196	29	Female	GYN	Less than six drugs	No	0	No	No	MALARIA IN PREGNANCY, VAGINAL CANDIDIASIS, UTI	EPIGASTRIC PAIN	DICLOFENAC
0.196	25	Female	GYN	Less than six drugs	No	0	No	No	MALARIA IN PREGNANCY	NAUSEA<br>WATERY NON-BLOODY DIARRHOEA<br>BLURRED VISION<br>TINNITUS<br>PALPITATIONS<br>DIZZINESS<br>TASTE DISTURBANCE & LOSS OF APPETITE	QUININE<br>QUININE<br>QUININE<br>QUININE<br>QUININE<br>QUININE<br>QUININE
0.196	30	Female	GYN	Less than six drugs	No	0	No	No	INTRAUTERINE FOETAL DEATH OF 22 WEEKS	LOWER ABDOMINAL PAIN<br>VOMITING	MISOPROSTOL<br>MISOPROSTOL
0.196	28	Female	GYN	Less than six drugs	No	0	No	No	BLIGHTED OVUM	DIARRHOEA	MISOPROSTOL
0.196	34	Female	GYN	Less than six drugs	No	0	No	No	MISSED ABORTION	DIARRHOEA<br>LOWER ABDOMINAL PAIN<br>VAGINAL BLEEDING	MISOPROSTOL<br>MISOPROSTOL<br>MISOPROSTOL
0.196	25	Female	GYN	Less than six drugs	No	0	No	No	MALARIA IN PREGNANCY	HEADACHE	QUININE
0.197	24	Female	GYN	Less than six drugs	No	0	No	No	MALARIA IN PREGNANCY	LOWER ABDOMINAL PAIN<br>VAGINAL BLEEDING	QUININE<br>QUININE
0.196	26	Female	GYN	Less than six drugs	No	0	No	No	INCOMPLETE MISCARRIAGE	LOWER ABDOMINAL PAIN<br>VAGINAL BLEEDING	MISOPROSTOL<br>MISOPROSTOL
0.196	29	Female	GYN	Less than six drugs	No	0	No	No	UTI IN PREGNANCY	HEADACHE	AMOXIL/CLAVULANATE
0.150	30	Female	CPN	Less than six drugs	No	1	No	Yes	HYPERTENSIVE HEART DISEASE, CONGESTIVE CARDIAC FAILURE	EPIGASTRIC PAIN<br>DIZZINESS	CAPTOPRIL<br>CAPTOPRIL & CARVEDILOL
0.101	32	Female	CPN	Less than six drugs	No	0	No	No	BRONCHO-PNEUMONIA, ASTHMA, ACUTE BRONCHITIS	HEADACHE	CEFTRIAXONE
0.146	26	Female	CPN	Six or more drugs	No	2	Yes	No	ANAEMIA IN IMMUNOSUPPRESSED SYNDROME (ISS), PULMONARY TUBERCULOSIS (PTB) RELAPSE, PNEUMONIA	FEVER<br>DIZZINESS<br>VOMITING	CEFTRIAXONE<br>CEFTRIAXONE<br>CEFTRIAXONE
0.056	85	Female	CPN	Less than six drugs	No	5	No	No	HYPERTENSION, ATRIAL FIBRILLATION, ISCHAEMIC HEART DISEASE, COMPLETE HEART BLOCK	COUGH	CAPTOPRIL
0.102	25	Female	CPN	Less than six drugs	No	0	No	No	PNEUMONIA, PTB	DIARRHOEA<br>FEVER<br>LOSS OF APPETITE	CEFTRIAXONE<br>CEFTRIAXONE<br>METRONIDAZOLE
0.100	29	Male	CPN	Less than six drugs	No	0	No	No	BRONCHO-PNEUMONIA, RULE-OUT (R/O) PTB, LOBAR PNEUMONIA	HEADACHE	CEFTRIAXONE

Probability - *possible* ADR	age	gender	ward	No of drugs administered	Herbal medicines	charlson's index	HIV-positive	Hospitalization	Working diagnosis	*Possible* ADR	Implicated drug
0.140	63	Female	CPN	Six or more drugs	No	3	No	No	DIABETES MELLITUS, HYPERTENSION, HYPERTENSIVE HEART DISEASE (HHT)	EPIGASTRIC PAIN	DIGOXIN, FRUSEMIDE
0.156	52	Male	CPN	Less than six drugs	Yes	1	No	No	LOBAR PNEUMONIA, ?PTB	DIARRHOEA<br>ABD PAIN	ERYTHROMYCIN<br>DICLOFENAC, ERYTHROMYCIN
0.103	40	Male	CPN	Less than six drugs	No	6	Yes	No	ISS, PTB, PNEUMONIA	VOMITING	CEFTRIAXONE
0.138	68	Male	CPN	Six or more drugs	No	3	No	No	TENSION PNEUMOTHORAX, LEFT HEART FAILURE	CONSTIPATION	TRAMADOL
0.101	22	Male	IDGI	Less than six drugs	No	0	No	No	SEPTICAEMIA SECONDARY TO UTI, ELECTROLYTE IMBALANCE	NAUSEA (REDUCED INFUSION RATE & ADR REDUCED)	CIPROFLOXACIN
0.162	55	Female	IDGI	Less than six drugs	Yes	8	Yes	No	ISS WITH GASTROENTERITIS (G/E), ORAL CANDIDIASIS, BRONCHOPNEUMONIA	NAUSEA	METRONIDAZOLE
0.102	18	Female	IDGI	Less than six drugs	No	0	No	No	SEVERE MALARIA, SEVERE ANAEMIA, ACUTE KIDNEY INJURY	VOMITING	PARACETAMOL
0.149	40	Female	IDGI	Less than six drugs	No	0	No	Yes	ORGANIC PSYCHOSIS, RESOLVING LYME MENINGITIS	BLURRED VISION<br>TREMORS	BENZTROPINE<br>HALOPERIDOL
0.149	28	Male	IDGI	Less than six drugs	No	0	No	Yes	ORGANOPHOSPHATE POISONING, ATTEMPTED SUICIDE	DROWZINESS	ATROPINE
0.145	34	Female	IDGI	Six or more drugs	No	0	Yes	No	ORAL CANDIDIASIS, UTI, R/O MALARIA	NAUSEA AND VOMITING	KETOCONAZOLE
0.102	56	Male	IDGI	Less than six drugs	No	7	Yes	No	ISS, MALARIA, SEPTICAEMIA, PTB, TUBERCULOSIS ADENITIS, LYMPHOMA	DIARRHOEA	CEFTRIAXONE
0.144	35	Male	IDGI	Six or more drugs	No	0	Yes	No	PERITONITIS, ACUTE PERSISTENT PANCREATITIS	SEVERE ABD PAIN	CIPROFLOXACIN
0.164	28	Female	IDGI	Less than six drugs	Yes	6	Yes	No	ISS, HYPOTENSION, ABDOMINAL TUBERCULOSIS, CHOLESTATIC DISEASE, UTI, MALARIA, MODERATE ANAEMIA	VOMITING	CEFTRIAXONE
0.146	27	Female	IDGI	Six or more drugs	No	0	Yes	No	ISS ON HAART & CO-TRIMOXAZOLE, CRYPTOCOCCAL MENINGITIS, G/E, MENINGOENCEPHALAITIS, ACUTE BACTERIAL MENINGITIS, TENSION HEADACHE	VOMITING	TRAMADOL
0.159	18	Male	HNE	drugs	Yes	0	No	No	ISS, BRONCHO-PNEUMONIA	DECREASED URINE OUTPUT	CEFTRIAXONE

Appendix S5: Characteristics of 35 patients with *possible* hospital-acquired suspected ADRs in the top 10% (high-risk group) of *possible* ADR risk distribution, Uganda, 2014

Probability - *possible* ADR	age	gender	ward	No of drugs administered	Herbal medicines	charlson's index	HIV-positive	Hospitalization	Working diagnosis	*Possible* ADR	Implicated drug
0.509	29	Female	GYN	Six or more drugs	No	1	No	Yes	PUEPERIAL SEPSIS, PELVIC ABSCESS	VOMITING	CEFTRIAXONE
0.511	21	Female	GYN	Six or more drugs	No	0	No	Yes	SEVERE MALARIA IN PREGNANCY, SEVERE ANAEMIA	VOMITING ALL FEEDS	QUININE
0.407	28	Female	GYN	Six or more drugs	No	6	Yes	No	INCOMPETENT CERVIX, THREATENED ABORTION	MILD RADIATING HEADACHE	OXYTOCIN
										RAISED PULSE	OXYTOCIN
0.525	41	Female	GYN	Six or more drugs	Yes	0	No	No	PELVIC INFLAMMATORY DISEASE, ENDOMETRIOSIS, PERITONITIS, PELVIC ABSCESS	NAUSEA	TRAMADOL & CEFTRIAXONE
										VOMITING	TRAMADOL & CEFTRIAXONE
0.518	27	Female	GYN	Six or more drugs	No	6	Yes	Yes	THREATENED ABORTION	VAGINAL BLEEDING WITH BLOOD CLOTS	MISOPROSTOL
										LOWER ABDOMINAL PAIN WITH SPASMS	MISOPROSTOL
0.528	21	Female	GYN	Six or more drugs	Yes	0	No	No	MALARIA IN PREGNANCY, BACTEREMIA	DECREASED APPETITE	ERYTHROMYCIN
										VOMITING	ERYTHROMYCIN
0.528	25	Female	GYN	Six or more drugs	Yes	0	No	No	INEVITABLE ABORTION, INCOMPLETE ABORTION	VOMITING	CEFTRIAXONE
0.636	24	Female	GYN	Six or more drugs	Yes	0	No	Yes	ANAEMIA SECONDARY TO SEVERE MALARIA, UTI IN PREGNANCY	CHILLS - MILD HYPERSENSITIVITY	BLOOD
										VOMITING	BLOOD
0.528	22	Female	GYN	Six or more drugs	Yes	0	No	No	THREATENED ABORTION	DYSURIA	NIFEDIPINE
0.400	18	Female	GYN	Six or more drugs	No	0	No	No	INCOMPLETE ABORTION	LOWER ABDOMINAL PAIN	OXYTOCIN
0.509	29	Female	GYN	Six or more drugs	No	0	No	Yes	POST-PARTUM HAEMORRHAGE, DYSFUNCTIONAL UTERINE BLEEDING, MODERATE ANAEMIA	ABDOMINAL PAIN	MISOPROSTOL
0.636	29	Female	GYN	Six or more drugs	Yes	0	No	Yes	?ELECTROLYTE IMBALANCE, ?SEPSIS	DIZZINESS	CEFTRIAXONE, METRONIDAZOLE
0.528	26	Female	GYN	Six or more drugs	Yes	0	No	No	INCOMPLETE ABORTION, MENORRHAGIA	VAGINAL BLEEDING	MISOPROSTOL
										PALPITATIONS	MISOPROSTOL
0.400	19	Female	GYN	Six or more drugs	No	0	No	No	INCOMPLETE ABORTION	VOMITING	AMPICILLIN, METRONIDAZOLE, CEFTRIAXONE, LEVOFLOXACIN
										MID LOWER ABDOMINAL PAIN	LEVOFLOXACIN
										DIZZINESS	METRONIDAZOLE, HALOPERIDOL
										LOSS OF APPETITE	LEVOFLOXACIN, MISOPROSTOL, METRONIDAZOLE, HALOPERIDOL
										HEADACHE	CIPROFLOXACIN, LEVOFLOXACIN
										NOT ORIENTED TO TIME & PLACE - POOR SPEECH	HALOPERIDOL
										FEVER	HALOPERIDOL
										LOW BLOOD PRESSURE	HALOPERIDOL
										STIFF NECK WITH SWOLLEN TONGUE	HALOPERIDOL
										UNCOORDINATED MOVEMENTS IN BED	HALOPERIDOL

Probability - *possible* ADR	age	gender	ward	No of drugs administered	Herbal medicines	charlson's index	HIV-positive	Hospitalization	Working diagnosis	*Possible* ADR	Implicated drug
0.527	30	Female	GYN	Six or more drugs	Yes	0	No	No	SEVERE ANAEMIA, ENDOMETRIOSIS, FIBROSIS	FEVER	IRON SUCROSE
										DIZZINESS	TRANEXAMIC ACID, IRON SUCROSE
										PALPITATIONS & EASY FATIGUABILITY	IRON SUCROSE
										EPIGASTRIC PAIN	FEFOL
										HEADACHE	IRON SUCROSE
										PAINFUL LOWER LIMBS	IRON SUCROSE
0.636	24	Female	GYN	Six or more drugs	Yes	1	No	Yes	POST OPERATIVE SEPSIS, RUPTURED ECTOPIC	VOMITING	LEVOFLOXACIN, CEFTRIAXONE
										LOSS OF APPETITE	LEVOFLOXACIN, CEFTRIAXONE
										ALLERGIC SKIN REACTION ON ARM	LEVOFLOXACIN, CEFTRIAXONE
										HEADACHE	LEVOFLOXACIN, CEFTRIAXONE, HALOPERIDOL
										GENERAL BODY WEAKNESS	LEVOFLOXACIN, DIAZEPAM
										DIZZINESS	DIAZEPAM, LEVOFLOXACIN, CEFTRIAXONE
0.510	22	Female	GYN	Six or more drugs	No	0	No	Yes	INCOMPLETE ABORTION	FEVER	CEFTRIAXONE
										BACK PAIN	MISOPROSTOL, OXYTOCIN
0.511	21	Female	GYN	Six or more drugs	No	1	No	Yes	SEVERE ANAEMIA	CONSTIPATION	FEFOL
										HEADACHE	MICROGYNON
										EPIGASTRIC PAIN	FEFOL
0.508	39	Female	GYN	Six or more drugs	No	1	No	Yes	MOLAR PREGNANCY, INCOMPLETE ABORTION	DIZZINESS	CEFTRIAXONE
0.637	22	Female	GYN	Six or more drugs	Yes	0	No	Yes	SEVERE ANAEMIA, SECONDARY MENORRHAGIA	HEADACHE	MICROGYNON
0.527	28	Female	GYN	Six or more drugs	Yes	0	No	No	CHRONIC LEAKING ECTOPIC PREGNANCY, THREATENED ABORTION	PARAESTHESIA	LIGNOCAINE
0.636	25	Female	GYN	Six or more drugs	Yes	0	No	Yes	INCOMPLETE ABORTION, MENORRHAGIA, MODERATE ANAEMIA	DIZZINESS	MISOPROSTOL
0.442	40	Male	CPN	Six or more drugs	Yes	0	No	Yes	ACUTE KIDNEY INJURY SECONDARY TO TOXINS FROM HERBS, GLOMERULAR NEPHRITIS, SEVERE ANAEMIA	VOMITING	CEFTRIAXONE, CAPTOPRIL, FRUSEMIDE
										DIARRHOEA	CEFTRIAXONE, FRUSEMIDE,CAPTOPRIL,CALCIUM GLUCONATE
										ABD PAIN	CEFTRIAXONE, CAPTOPRIL, FRUSEMIDE
0.448	19	Female	CPN	Six or more drugs	Yes	0	No	Yes	SEVERE PNEUMONIA, SEPTICAEMIA, POORLY MANAGED MALARIA, PULMONARY TUBERCULOSIS (PTB)	CONSTIPATION	METRONIDAZOLE
										SHORTNESS OF BREATH	ISONIAZID, RIFAMPICIN, PYRAZINAMIDE, ETHAMBUTOL (HRZE)
0.444	28	Male	CPN	Six or more drugs	Yes	0	No	Yes	HYPERTENSION, CHRONIC KIDNE DISEASE, ACUTE KIDNEY INJURY	OEDEMA	CARVEDILOL
0.439	61	Male	CPN	Six or more drugs	Yes	2	No	Yes	COR PULMONALE, ACUTE SEVERE ASTHMA	RAISED SYSTOLIC BLOOD PRESSURE	AMINOPHYLLINE
0.442	60	Female	CPN	Six or more drugs	Yes	2	No	Yes	LUNG MALIGNANCY	DIZZINESS	CEFTRIAXONE, TRAMADOL
										PARAESTHESIA	CEFTRIAXONE
										CONSTIPATION	TRAMADOL

Probability - *possible* ADR	age	gender	ward	No of drugs administered	Herbal medicines	charlson's index	HIV-positive	Hospitalization	Working diagnosis	*Possible* ADR	Implicated drug
0.443	33	Male	HNE	Six or more drugs	Yes	0	No	Yes	SEVERE ANAEMIA, MALARIA	VOMITING	BLOOD
										SEVERE HEADACHE	BLOOD
										BLEEDING FROM THE GUM	BLOOD
										VOMITING	BLOOD
0.446	32	Female	HNE	Six or more drugs	Yes	0	No	Yes	ANAEMIA, MALARIA, ? UPPER GASTROINTESTINAL BLEEDING	VOMITING	LUMARTEM, CIPROFLOXACIN
0.442	59	Female	HNE	Six or more drugs	Yes	1	No	Yes	SEVERE ANAEMIA	VOMITING	LEVOFLOXACIN, CYPROHEPTADINE
										GENERAL BODY WEAKNESS	LEVOFLOXACIN, CYPROHEPTADINE
0.448	20	Female	HNE	Six or more	Yes	0	No	Yes	SICKLER IN VASOCCLUSIVE CRISIS	JAUNDICE	BISACODYL
0.443	36	Male	IDGI	Six or more drugs	Yes	1	No	Yes	SEVERE MALARIA, HEPATITIS, UTI	VOMITING	COARTEM
0.455	29	Female	IDGI	Six or more drugs	Yes	7	Yes	Yes	HIV COMPLICATED WITH CCM & TUBERCULOSIS	VOMITING	TRAMADOL
										MALAISE	AMPHOTERICIN B
0.444	28	Male	IDGI	Six or more drugs	Yes	0	No	Yes	CHRONIC ABD PAIN, INFLAMMATORY BOWEL DISEASE, HAEMORRHOIDS, PANCREATITIS, AORTIC DISSECTION	TACHYCARDIA	CIPROFLOXACIN
										HYPERTENSION	MORPHINE
0.452	45	Female	IDGI	Six or more drugs	Yes	7	Yes	Yes	IMMUNOSUPPRESSED SNDROME (ISS),CHOLELITHIASIS, STEATOSIS OF LIVER, TB IRIS, ACUTE LIVER INJURY, DRUG-INDUCED HEPATITIS,SOME DEHYDRATION, VIRAL HEPATITIS, SEPSIS, CRYPTOCOCCAL MENINGITIS	HYPOVOLAEMIA	FRUSEMIDE
